# Factors Influencing Telehealth Use in School-based Health Services: Secondary Analysis from a Scoping Review

**DOI:** 10.63144/ijt.2025.6652

**Published:** 2025-06-12

**Authors:** Erin Knobl, Kari Renahan, Annie Jiang, Michelle Phoenix, Briano Di Rezze, Wenonah Campbell

**Affiliations:** 1 School of Rehabilitation Science, McMaster University, Hamilton, Canada; 2 CanChild, McMaster University, Hamilton, Canada

**Keywords:** Children, Diffusion of innovations, Implementation, School, Scoping review

## Abstract

**Introduction:**

Although telehealth use in schools can address gaps in service access, implementation in the school setting lags. This study describes factors that influence implementation of telehealth in school health services.

**Methods:**

A protocol was published *a priori*. Using scoping review methods, articles were sought in five academic databases pertaining to regulated health providers’ use of telehealth in kindergarten to grade 12 schools. Two reviewers completed source selection and data extraction. Data were charted to the diffusion of innovations theory and content analysis performed.

**Results:**

Of 6585 unique sources considered, 70 articles were included. Multiple factors were described influencing telehealth implementation in schools. The most salient factors reported for successful implementation included provider training, access to reliable technology, availability of an e-helper, and policies to support ethical telehealth delivery.

**Conclusion:**

Telehealth use in schools is increasing; however, successful implementation requires planning that considers how and why such innovations are adopted.

Telehealth is the provision of health care using telecommunications to reach patients at a distance (Nickelson, 1998). In a school setting, telehealth may be used to address staff shortages, to serve rural students, and to abide by COVID-19 school closures (Knobl et al., 2024). Telehealth use in schools had been growing prior to the COVID-19 global pandemic; however, with the pandemic restrictions and school closings, telehealth use has increased dramatically (Grogan-Johnson, 2021). For example, prior to the COVID-19 pandemic, a survey of school-based speech-language pathologists (SLPs) in one region of the United States (US) found that just 2.4% of respondents had previously used telehealth, and only 1.8% were using it at the time of the 2012 survey (Tucker, 2012). However, a national US survey conducted during the COVID-19 pandemic, found that 60% of school-based SLPs were using telehealth for all or some of their caseload (Tambyraja et al., 2021). Similarly, pediatric physical therapists (PTs) practicing in outpatient, early intervention, and school-based settings, across the US reported no use or training in telehealth prior to the pandemic, but 61.9% of respondents were using telehealth for more than half their caseload during the pandemic (Hall et al., 2021). Likewise, psychologists working in school practice in Australia shifted their service provision during the pandemic; 86% of school psychologists used videoconferencing and 61% used telephone counselling (Hyde et al., 2022) as compared with only 43% of psychologists using telehealth previously (Glueckauf et al., 2018). Furthermore, as the pandemic subsides, continued use of telehealth is recommended to better meet the needs of some families and children or to supplement in-person services (Goddard et al., 2021; Rosenbaum et al., 2021; World Health Organization, 2021a, 2021b).

As noted, even prior to the global pandemic, some school-based health providers were interested in delivering services using telehealth. To illustrate, Tucker (2012) reported that 31.2% of surveyed school-based SLPs from one northeastern US state were interested in providing telehealth, 26.5% were neutral in their interest in telehealth, and 42.4% did not want to provide telehealth. In 2019, Rortvedt and Jacobs reported that among occupational therapists (OTs) in the midwestern United States, 28.57% believed their district was likely to adopt telehealth. The use of telehealth in schools during the pandemic was described as emergency use that was implemented without preparation or training as a sudden shift in service delivery (Grogan-Johnson, 2021). Grogan-Johnson (2021) argued that this emergency pandemic use is not the same as implementing telehealth as a service delivery model for sustained use beyond the pandemic and that intentional implementation can improve how school-based services meet the needs of students.

## Telehealth Implementation

The limited pre-pandemic use of telehealth by school health providers is not surprising when one considers that ideas present in the health literature generally have a low and slow uptake into practice, ranging from 14–50% of health research being implemented into practice with a recognized lag of approximately 17 years (Balas & Boren, 2000; Z. S. Morris et al., 2011). Models of acceptance of health technology are often missing the temporal aspect of implementing health technology (Nadal et al., 2020). The lag between health technology innovation and health implementation may be costly to the health system if services are withheld while waiting for further evidence to support a new technology or while waiting for implementation plans to be developed (Z. S. Morris et al., 2011). For example, if telehealth service delivery in schools is deemed effective, then delayed implementation in an underserved area where children await services on a waitlist will likely result in negative compounding effects. Furthermore, oftentimes, research studies are conducted in a controlled setting and when completed, there is no further follow up for implementation (Kristensen et al., 2016). However, implementation science researchers are now aware of and are attempting to close the research to practice gap (Kristensen et al., 2016).

Additionally, initial acceptance or rejection of a health technology does not necessarily correlate to later sustained use of health technology (Nadal et al., 2020). For example, a health provider may initially decide to use telehealth to deliver services, and then after using it for a period, may decide to return to exclusively providing in-person services. Many reasons exist to initiate or terminate use of a service delivery model, such as clinical decision making, individual preferences, and resource availability (Camden & Silva, 2021). This complicates the ability to measure implementation and use of health technology as it may fluctuate.

Implementation research has identified that sharing knowledge through guidelines and instructions for clinicians is not sufficient for achieving implementation results (Campbell et al., 2020; Kirchner et al., 2020; Kristensen et al., 2016). A thorough multifaceted plan is needed to implement research into practice (Greenhalgh et al., 2017; Grimshaw et al., 2012; Grol, 1997; Kho et al., 2020; Kirchner et al., 2020). Part of planning for implementation of novel, clinically relevant research is to understand and identify potential barriers. These barriers may exist at the individual clinician level, where there is a need to understand and believe in the change (Beauchemin et al., 2019); at the team level, where there is a need for a team leader to take responsibility for driving the change action (Kristensen et al., 2016); and at the organisational level, where different people interact and depend on each other to create conditions for change (Grol, 1997; Grol & Grimshaw, 2003). Implementation science often targets the individual level (Colquhoun et al., 2017) and little information is known about targeting middle managers and policy makers (Grimshaw et al., 2012; Kristensen et al., 2016). Nonetheless, it is recommended that a healthcare implementation plan should include identification of barriers and use theory (Colquhoun et al., 2017; Grimshaw et al., 2012).

## Diffusion of Innovations

Diffusion of innovations (DOI) is a theory used to explain spread, adoption, and implementation of novel ideas (Rogers, 2013). DOI is well established and used in a variety of research studies (Colquhoun et al., 2010). Developed by Everett Rogers in agriculture studies in the 1940’s, DOI has spread to fields of communication studies and public health, and widely to numerous other academic disciplines (Rogers, 2003). Examples of the application of DOI in research exist in telehealth studies (Dimmick & Ignatova, 2006; Haun et al., 2020; Peeters et al., 2012; Sugarhood et al., 2014; Zhang et al., 2015) and school health initiatives (Audrey et al., 2004; Glowacki et al., 2016; Harriger et al., 2014; Naylor et al., 2016; Radis et al., 2016; Steckler et al., 1992). A review of use of theory in scoping reviews identified 27 articles in rehabilitation, nursing, and medicine that applied DOI in their research (Colquhoun et al., 2010), reporting that DOI was used in health research to address general innovations (e.g., research uptake) as well as specific innovations (e.g., a particular intervention or assessment) and was commonly used to assess potential predictors of behaviour change.

DOI describes factors that affect the process of adoption of a new idea into a society, such as the implementation of telehealth in schools. This theory comprises four main elements as well as many sub elements. Briefly, the four main elements include: innovation, communication channels, time, and social system. The innovation refers to the new idea or product and has several characteristics that aide in its path to adoption. These characteristics are relative advantage, compatibility, complexity, trialability, observability, and re-invention.

The second element, communication channels, refers to the pathway through which an individual learns about an innovation. These pathways can be formal or informal and can include mass media.

The third element, time, is about the process and steps taken towards adoption. Two groupings of related concepts exist within time. The first, the innovation-decision process is the steps an individual takes starting with gaining knowledge (learning about the innovation), persuasion (forming an opinion about the innovation), decision, implementation, and confirmation. The second, the adopter categories, which includes notions of who within a group of people adopts the novel innovation first (innovators) through to who adopts the novel innovation last (laggards).

Finally, the social system is the fourth element, which includes the people within a group that affect and are affected by the implementation of an innovation. The social system also encompasses two groupings of related concepts. The first focuses on how a decision to adopt an innovation is made whether by an individual (optional), a collective (e.g., vote), or by a person of authority. The second discusses the consequences of the innovation. These refer to the desired and undesired, direct and indirect, and anticipated and unanticipated changes that happen within a society based on the adoption of an innovation. For further definitions and descriptions, please see Rogers’ *Diffusion of Innovations* (Rogers, 2003).Together, the four elements of DOI theory and their respective sub elements explain the factors that influence adoption of an innovation. The elements of DOI can be used to gain an understanding of the implementation of telehealth use in the school context, including the implementation facilitators and barriers.

## Current Review

Given that telehealth use in school-based services was increasing prior to the global pandemic, and the pandemic has provided health providers, educators, students, and families an opportunity to trial telehealth, it is likely that telehealth as a service delivery model will continue after the pandemic. For these reasons, we were interested in gaining a broader understanding of telehealth use in schools. We previously completed a companion scoping review of the characteristics of telehealth use in schools and described what telehealth looks like in schools, who uses telehealth, when telehealth is used, and why telehealth is used (Knobl et al., 2024). Using the same articles, we now conduct a secondary analysis to further explore telehealth use in schools by identifying factors that affect telehealth use in school-based health services in kindergarten to grade 12 schools.

## Methods

We followed the *JBI Manual for Evidence Synthesis* (Peters, Godfrey, et al., 2020) and the “Preferred Reporting Items for Systematic Reviews Extension for Scoping Reviews (PRISMA-ScR): Checklist and Explanation” (Tricco et al., 2018) when completing this review. A protocol was published prior to initiating the search strategy (Knobl et al., 2021). As this review was conducted as a secondary analysis to an initial scoping review on the characteristics of telehealth used in school-based health services, more detailed methods can be found in that paper (Knobl et al., 2024).

### Inclusion Criteria

We included peer-reviewed articles, published in English, and available through our university’s health science library databases or open-source material. We did not date limit the year of publication. Included articles needed to pertain to regulated health professionals who were providing telehealth in a school setting. The population criteria included services provided or supervised by a regulated health professional. A regulated health professional is a health professional who is required to be a member of a regulatory body or licensing board that ensures a standard of service that benefits the public (Health Profession Regulators of Ontario, 2023). The inclusion criterion for telehealth allowed for any service (e.g., assessment, consultation, meeting, intervention) to be provided by telehealth and for the telehealth to be provided using synchronous or asynchronous technology. Finally, the school context criterion focused on the telehealth service being integrated within any kindergarten to grade 12 school with the focus of services on increasing access to the educational curriculum. This criterion limited purely medical model services that did not have an educational outcome (e.g., a primary health clinic housed in a school).

### Search Strategy

A search strategy was developed with the guidance of a university health sciences librarian. We used the inclusion criteria pertaining to regulated health professionals, telehealth, and school context to develop a list of synonyms for use as keywords and MeSH headings. The search was conducted in five academic databases: Cumulative Index to Nursing and Allied Health Literature (CINAHL), Embase, Education Resources Information Center (ERIC), MEDLINE, and APA PsycInfo and includes articles up to January 18, 2024. Additionally, we reviewed the reference lists of included articles to locate additional sources. See the Appendix for a detailed list of search terms and a sample search.

### Sources of Evidence Selection

All duplicates were removed by the first author, first automatically using EndNote, then manually using a visual screening in EndNote, and finally automatically using Covidence software (Veritas Health Innovation, 2022). Covidence (Veritas Health Innovation, 2022) was used for the remainder of the source selection. All sources were screened at title and abstract by the first author and one additional reviewer (KR, AJ, HA, or CT) following completion of training and piloting to achieve consistency in interpretation of inclusion criteria. Any disagreements for inclusion were discussed between the two conflicting reviewers to obtain consensus. Following title and abstract screening, full-text screening was undertaken in a similar fashion.

### Data Extraction, Analysis and Presentation of Results

Scoping review methodology recommends descriptive qualitative content analysis and use of an a priori framework (Levac et al., 2010; Peters, Godfrey, et al., 2020; Peters, Marnie, et al., 2020; Pollock et al., 2022). Deductive content analysis was conducted following the methods outlined in Elo and Kyngäs (2008), and proceeded in three phases; preparation, organization, and reporting.

In the first phase, preparation, the first author developed a data extraction chart based on the DOI theory (Rogers, 2003). Although Rogers (2003) uses the term elements to describe the four main elements of diffusion, we will use the terms categories and subcategories to align with qualitative content analysis methods (Elo & Kyngas, 2008). Initially, the extraction chart included only the four main categories of DOI (innovation, communication channels, time, and social system). Subsequently, after protocol publication, the data extraction chart was expanded to include the more narrowly focused layer of subcategories. For example, instead of extracting to the broad category of innovation, the modified data extraction chart breaks innovation into six subcategories: relative advantage, compatibility, complexity, trialability, observability, and re-invention. The narrower subcategories are taken from the DOI theory and were included for the elements of innovation, time, and social system to allow for more specificity in reporting results. Communication channels did not have further subcategories within DOI to which we could extract. The definitions used for each of the categories and subcategories were taken verbatim from Chapter 1: Elements of diffusion, with additional details sought in later chapters as needed (Rogers, 2003). Additionally, the unit of meaning was selected as any verbatim segment [phrase, sentence(s)] that fit within the subcategories to be extracted.

In the second phase, organization, the reviewers familiarized themselves with the included articles and the data extraction form. The revised data extraction chart was trialed by the team (first, second, and third authors) using three included articles. During the trial, additional categories were added to allow for coding of data that did not fit within the DOI framework. These included a subcategory under each of innovation, time, and social system, as well as three additional categories to capture other barriers, facilitators, and factors that influence telehealth use not addressed by the DOI theory. Table 1 shows the data extraction template. Following trialling of the data extraction form, the remainder of the articles were extracted by the first author and verified by another reviewer (second or third author). Any changes to the article extractions were determined by consensus with regular team check-ins taking place as needed. Memoing was used to organize the data within each subcategory, with additional consultations with team members experienced in qualitative methods (fourth and sixth authors).

In the third phase, reporting, the first author organized the categories and subcategories to which data were charted. Visual and descriptive representation of coded data were developed for presentation of the results (Lockwood et al., 2019; Peters, Godfrey, et al., 2020).

## Results

### Search

**Figure 1:**
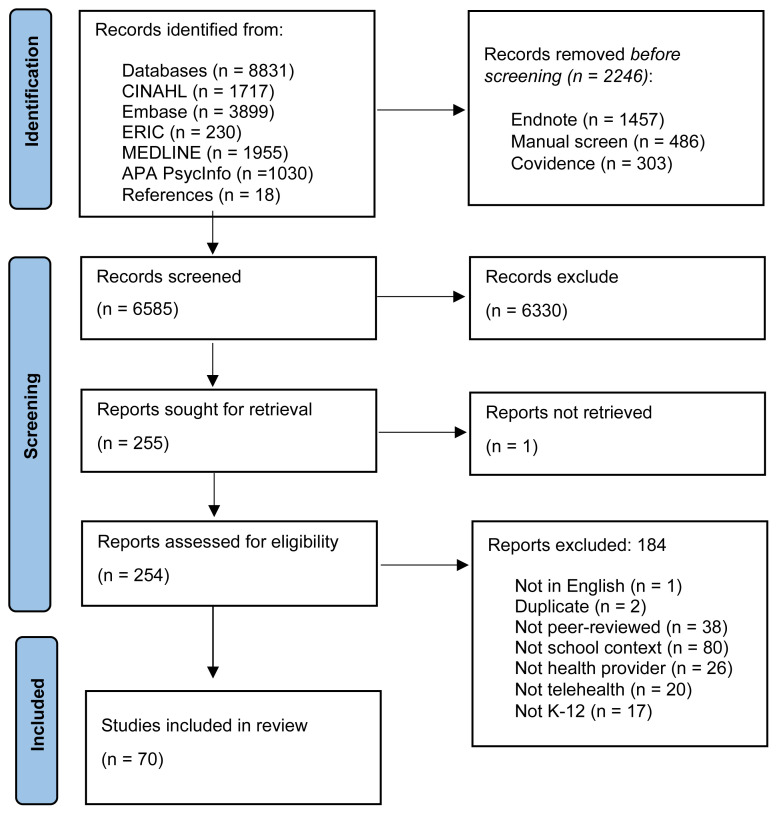
PRISMA Flow Chart for Source Selection *Note*. Reprinted from “A scoping review of telehealth in school-based health services: Characteristics of telehealth use” by Knobl et al., 2024 (https://doi.org/10.31219/osf.io/t2chx)

Five academic databases and reference lists of included articles were searched through January 18, 2024, to identify 6585 unique sources. Full text review of 254 sources was completed and 70 articles were included for data extraction (Figure 1). Training and piloting of inclusion criteria was completed with all reviewers until minimum 80% agreement was achieved as set in the protocol (Knobl et al., 2021). Included articles were not date limited and spanned from 1998 thru 2023, though nearly three-quarters of the articles had been published since 2017 (n=51, 73%). A majority (n=47, 67%) originated from the United States and were empirical studies (n=54, 77%) of various methodologies and design types. See results section of the prior scoping review paper for further details on source selection and source characteristics (Knobl et al., 2024).

### Diffusion of Innovations

**Figure 2:**
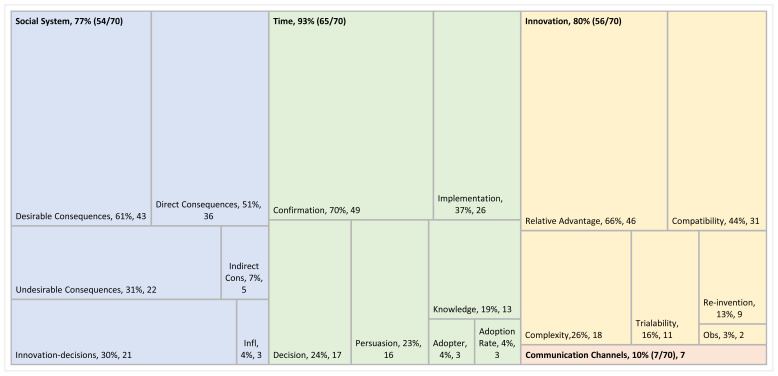
Percentage Distribution of Diffusion of Innovation Categories and Subcategories Across Included Articles *Note*. Obs = Observability. Adopter = Adopter Categories. Indirect Cons = Indirect Consequences. Infl = Influencers. Percentages reported followed by n of 70 articles.

The four main categories of the DOI theory – innovation, communication channels, time, and social system – were all represented by the included articles. However, these categories and subcategories were not represented equally across sources. Figure 2 illustrates proportionally how many of the articles addressed the main categories of DOI along with the subcategories; innovation (80%), time (93%), and social system (77%) categories are relatively equally represented compared to a smaller proportion for communication channels (10%). Additionally, Figure 2 demonstrates visually that subcategories are not represented equally in the literature; for example, relative advantage and compatibility are two of six subcategories within the innovation category but represent more than half of the extractions pertaining to the innovation. Table 2 shows which of each of the 70 included articles contained extractions pertaining to each of the subcategories.

### Innovation

The innovation, telehealth in school-based health services, is the first category of DOI and includes six subcategories: relative advantage, compatibility, complexity, trialability, observability, and re-invention (Rogers, 2003). Data were extracted to each DOI subcategory. In general, the positive characteristics of telehealth included increased access to services, decreased travel time for providers, and compatibility with the processes, steps, and clinical reasoning utilized for in-person services. The challenging characteristics of telehealth included a need for training in this different delivery method, decreased collaboration with education staff, and unavailable assessment validity standards for telehealth administration. Trialability and observability of telehealth were minimally reported within the literature.

#### Relative Advantage

The most frequently extracted subcategory within the innovation category was relative advantage (n = 46, 66%). Within these articles, the relative advantages of telehealth all involved comparisons between telehealth and traditional in-person service provision, with the exception of one article that reported videoconferencing as having a greater sense of presence than telephone-based telehealth (Rose et al., 2000). Of the relative advantages of telehealth, 18 articles discussed enhanced access to rural locations where there are frequently provider shortages (Bice-Urbach & Kratochwill, 2016; Bloomfield et al., 2020; Cox, 1998; Cox et al., 2000; Drabarek et al., 2022; Fairweather et al., 2017; Fischer, Bloomfield, et al., 2019; Fischer et al., 2018; Gabel et al., 2013; Lowman & Kleinert, 2017; McLellan et al., 2017; Miller et al., 2002; Nelson et al., 2023; Shahidullah et al., 2023; Tambyraja et al., 2021; Tucker, 2012; Walker et al., 2021; Zhang et al., 2023). Nine articles discussed provider advantages such as savings in travel time and better management of caseloads because of the time savings and increased flexibility allowed for by the telehealth technology versus in-person service provision (Abbott-Gaffney & Jacobs, 2020; Boisvert & Hall, 2019; Bolden & Grogan-Johnson, 2022; Fischer et al., 2016; Fischer, Dart, et al., 2019; Fischer, Dart, et al., 2017; Fischer et al., 2018; Rortvedt & Jacobs, 2019; Stephan et al., 2016). This time savings and caseload management advantage were also noted in eight articles to be advantages for the school system with cost savings gained through service efficiency and reduced payment for travel (Abbott-Gaffney & Jacobs, 2020; Craig et al., 2023; Fischer et al., 2016; Hall, 2022; Lowman & Kleinert, 2017; Machalicek et al., 2009; Schultz et al., 2018; Stormshak et al., 2019; Tucker, 2012). Eleven articles reported advantages for the student or family, including improved attention and engagement of the student when using a telehealth platform (Boisvert et al., 2012; Bolden & Grogan-Johnson, 2022; Nelson et al., 2023; Tucker, 2012) and family advantages, such as reduced travel time and ability to attend remotely from work or home (Gallagher, 2004; Green et al., 2023; Grogan-Johnson, 2021; McLellan et al., 2017; Stephan et al., 2016; Svendsen et al., 2023; Zhang et al., 2023). Finally, there were two reports of the advantages of the technology itself, including being able to record and save material for later asynchronous viewing (Fischer et al., 2018; Gallagher, 2004).

#### Compatibility

The compatibility of telehealth to the values, past experiences, and needs of the health providers, was the second most frequently coded subcategory of the innovation category (n = 31, 44%). Nine articles mentioned compatibility for providers following similar steps, processes, and clinical reasoning whether services are provided via telehealth or in-person (Boisvert et al., 2012; Bolden & Grogan-Johnson, 2022; Grogan-Johnson et al., 2011; Lundblom et al., 2022; Marrapese et al., 2021; McLellan et al., 2017; Stephan et al., 2016; Stormshak et al., 2019; Walker et al., 2021). One article described telehealth as being similar to a pull-out therapy model where the student is removed from the general education classroom and taken to a separate room for therapy (Grogan-Johnson et al., 2011). However, this article also noted that this is not the usual model of service delivery for school-based services due to difficulties with collaboration and linking the therapy intervention to the classroom curriculum (Grogan-Johnson et al., 2011).

A lack of compatibility between telehealth services and traditionally provided in-person services was mentioned in 10 articles. Specifically, cited incompatibilities included that the services were provided through a different delivery method (Bolden & Grogan-Johnson, 2022; Fischer et al., 2016; Gallagher, 2004; Grogan-Johnson, 2021), that there was a lack of interaction and difficulties with collaboration (Bolden & Grogan-Johnson, 2022; Daftary, 2021; Tambyraja et al., 2021; Tucker, 2012), and a lack of physical contact between the provider and the student which resulted in difficulties with demonstrating skills and providing hands on assistance for tasks (Abbott-Gaffney & Jacobs, 2020; Fischer, Bloomfield, et al., 2019; Rortvedt & Jacobs, 2019). Providers also expressed several concerns including the ability of telehealth to meet their service delivery needs while also meeting their professional values (Fischer et al., 2018; Grogan-Johnson, 2018; Tucker, 2012), child engagement during telehealth (Drabarek et al., 2022; Erickson et al., 2021; Farmer et al., 2020; Svendsen et al., 2023), and the quality and availability of the technology (Fischer et al., 2018; Grogan-Johnson et al., 2011; Rose et al., 2000; Tambyraja et al., 2021) when offering services via telehealth.

Validity and standardization of assessments is another concern identified for compatibility of telehealth service delivery (Berger et al., 2023; Brunson McClain et al., 2021; Farmer et al., 2020; Tucker, 2012). The standardization of assessment concerns are due to the uncontrolled environment when using telehealth that may include additional distractions not usually present during in-person assessment delivery, that the student may not be able to engage in the virtual setting, and/or the examiner may not be able to administer and view the response of the student the same as when in person (Farmer et al., 2020). Finally, there were statements that telehealth is an acceptable alternative given the context where services were provided such as rural locations and where demand for services exceeds the available workforce (Boisvert & Hall, 2019; Bolden & Grogan-Johnson, 2022; Cox, 1998; Criss, 2013; Fischer et al., 2016; Nelson et al., 2023; Rose et al., 2000; Stormshak et al., 2019; Tucker, 2012).

#### Complexity

Health providers reported needing training in the use of technology for providing services (Bloomfield et al., 2020; Boisvert & Hall, 2019; Craig et al., 2023; Frederick et al., 2020; Lam et al., 2023; Lundblom et al., 2022; Moirangthem & Ojha, 2022; Tucker, 2012) as well as how to troubleshoot and support educators and families in their role in the receipt and support of telehealth (Brunson McClain et al., 2021; Farmer et al., 2020; Nelson et al., 2023; Schultz et al., 2018; Zhang et al., 2023). Providers also needed to ensure that the home or school environment was set up and secured for safety of the child receiving telehealth as well as set up a safety plan for crisis response (Nelson et al., 2023; Shahidullah et al., 2023). Providers sought guidance on the selection and purchase of hardware and software given their predicted use of the technology, the changing nature of technology, and the need for privacy and confidentiality to be maintained (Fischer et al., 2018; Grogan-Johnson, 2018; Hall, 2022; Reupert, Greenfeld, et al., 2022; Schultz et al., 2018). Providers also stated a desire for guidance on the selection of which students were best suited for telehealth services (Lam et al., 2023; Tucker, 2012).

#### Observability and Trialability

Only two articles reported the ability to observe telehealth as a starting point to implementing telehealth (Erickson et al., 2021; Grogan-Johnson, 2018). No articles reported on observing the outcomes of telehealth. Six articles discussed trialling telehealth prior to full implementation of the telehealth service delivery; these included trialling at the student (Grogan-Johnson, 2021), provider (Tucker, 2012), or school level (Hall, 2022; Stephan et al., 2016), or trialling for the purposes of research (Fischer et al., 2016). One article made specific mention that providers had no previous telehealth experience (Hall et al., 2021). One program did a slow rollout of telehealth to inform future telehealth implementation and service delivery (Nelson et al., 2023). The pandemic was also discussed as a trial period for providers (Lam et al., 2023) and health professional students learning during the pandemic may be more adept to deliver psychological assessments using telehealth (Berger et al., 2023). Finally, there was mention of utilizing free trials of software to determine if it met the user’s needs (Grogan-Johnson, 2018).

#### Re-invention

Nine of 70 articles discussed that providers adapted and used the technology differently once they had implemented it. Re-invention was guided by improvements, changes, and purchases of new technology (Boisvert et al., 2012; Grogan-Johnson, 2021). These technology purchases along with the experience using telehealth allowed for providers to increase and expand the services offered within the schools (Hyde et al., 2022; Marrapese et al., 2021; Stephan et al., 2016). Additionally, as telehealth was implemented, users were able to adapt the implementation to meet the needs of local sites (Shahidullah et al., 2022), individual students (Frederick et al., 2020; Grogan-Johnson, 2021; Hall, 2022), and improve rapport and connection (Drabarek et al., 2022).

### Communication Channels

Communication channels, the second category of DOI, includes how people learn about telehealth in schools. Communication channels data were reported within only seven studies (Crutchley & Campbell, 2010; Hall, 2022; Lam et al., 2023; Lowman & Kleinert, 2017; Norman et al., 2021; Tambyraja et al., 2021; Zhang et al., 2023). Channels initiating from the health provider were reported twice in one article and both times were done through written communication from the health provider either to family or to teachers (Crutchley & Campbell, 2010). In two articles, health providers were described as seeking more information either from peers, professional associations, regulators, literature, or seminars with preference for intraprofessional discussions (Hall, 2022; Lam et al., 2023). One article put the onus on university programs to train providers in telehealth (Lowman & Kleinert, 2017). Finally, three articles referred to the absence of communication channels, including a lack of communication for idea sharing between schools (Norman et al., 2021), informing parents about telehealth (Tambyraja et al., 2021; Zhang et al., 2023), and providers communicating to administrators novel information about telehealth (Lowman & Kleinert, 2017).

### Time

Time, the third category of DOI, refers to the period between people first learning about telehealth through to their adoption of telehealth. The category includes the innovation-decision process (knowledge, persuasion, decision, implementation, and confirmation), the adopter categories (innovators, early adopters, early majority, late majority, and laggards), and the rate of adoption. The innovation-decision process demonstrated that knowledge was gained through training, providers experienced persuasion and developed positive or mixed views of telehealth, decisions to use telehealth were often due to provider shortages or pandemic restrictions, and implementation involved getting technology and an onsite adult helper ready. After implementation, the literature suggests that confirmation was obtained through students, providers, parents, and educators finding telehealth in schools to be satisfactory. However, adoption of telehealth prior to the pandemic occurred at a slow rate. The adopter categories were poorly reported on in the literature.

#### Knowledge

Knowledge was discussed in nine articles as being desired or received by providers through professional trainings (Abbott-Gaffney & Jacobs, 2020; Boisvert et al., 2012; Bolden & Grogan-Johnson, 2022; Erickson et al., 2021; Grogan-Johnson, 2021; Rortvedt & Jacobs, 2019; Stephan et al., 2016; Stormshak et al., 2019; Walker et al., 2021). Additional discussion of training included one study reporting that providers need to educate school administrators and parents about the uses and opportunities for telehealth (Lowman & Kleinert, 2017). Moreover, 16 articles were influenced by the increase of telehealth use due to the COVID-19 pandemic, where there were contradictory reports of whether most providers had received training (Tucker, 2012) or that due to the rapid implementation of telehealth during the pandemic, providers did not have time for orientation and training in telehealth (Grogan-Johnson, 2021). Lastly, two articles mentioned that by offering training, providers are more likely to adopt telehealth (Abbott-Gaffney & Jacobs, 2020; Rortvedt & Jacobs, 2019).

#### Persuasion

Attitudes towards telehealth were mostly reported as either positive (n = 6) or mixed (n = 7). Positive attitudes were excitement and confidence, and seeing services as desirable, beneficial, and having increased opportunities (Abbott-Gaffney & Jacobs, 2020; Boisvert et al., 2012; Cox et al., 2000; Crutchley & Campbell, 2010; Hall et al., 2021; Pahwa et al., 2021). Mixed attitudes consisted of some of the same positive attitudes but with the addition of another attitude held by the group under study. When mixed attitudes were reported, these included varied interest in implementing telehealth from both providers and school districts with concerns about implicit biases, ability to develop relationships, and feelings of apprehension and uncertainty (Erickson et al., 2021; Frederick et al., 2020; Grogan-Johnson, 2021; Hall, 2022; Hines et al., 2015; Lowman & Kleinert, 2017; Rortvedt & Jacobs, 2019). One article presented a more neutral view with therapists reflecting on the best service for their students, having neutral attitudes towards telehealth, and majority being undecided about implementing telehealth (Tucker, 2012). Articles presented the attitudes towards telehealth implementation of health care providers, school districts, and educators, but not of parents or students.

#### Decision

The decision category provides insights into when telehealth use is considered, the needs of the implementers, and the various decisions made. Prior to the pandemic, telehealth was only considered by school districts when there was a provider or service shortage (Fairweather et al., 2017; Lowman & Kleinert, 2017). The decision to implement telehealth included assessing the resource needs of the school (Grogan-Johnson, 2021) and the home, and the youths’ needs and developmental stage (Nelson et al., 2023). During the COVID-19 pandemic specifically, implementation varied by provider, ranging from not using telehealth to utilizing telehealth for 80–90% of the workday (Daftary, 2021), and by country, with Australian and German school psychologists providing more telehealth than Canadian and American school psychologists (Reupert, Schaffer, et al., 2022). Another survey found that some providers used telehealth, whereas others sent assignments home, or did not provide services during school closures (Murphy et al., 2021). Similarly, 66 of 72 SLPs used telehealth; five of those did not use telehealth because there was no school policy and one did not because of privacy concerns (Lam et al., 2023). Decisions to use telehealth were more readily made at the school level by independent schools as compared with government schools that waited for guidelines to be developed (Reupert, Greenfeld, et al., 2022).

#### Implementation

The implementation phase was discussed in 12 articles and consists of determining technology needs, distributing technology, and setting up technology (Boisvert & Hall, 2019; Erickson et al., 2021; Fischer, Bloomfield, et al., 2019; Fischer et al., 2016; Fischer, Dart, et al., 2017; Frederick et al., 2020; Hall, 2022; Lee et al., 2017; Lincoln et al., 2015; Rose et al., 2000; Stephan et al., 2016; Walker et al., 2021). In addition to these technology specific implementation components, eight articles also discussed engaging and training resource personnel, such as an e-helper, who is on-site and whose role is to connect the student to the telehealth technology, trouble-shoot any technology issues, and provide on-site adult supervision during telehealth (Fairweather et al., 2017; Gabel et al., 2013; Grogan-Johnson et al., 2011; Hines et al., 2015; Nelson et al., 2023; Shahidullah et al., 2022; Shahidullah et al., 2023; Walker et al., 2021). Finally, as a first step to commencing telehealth, providers must obtain consent to deliver services (Fischer, Bloomfield, et al., 2019; Marrapese et al., 2021).

#### Confirmation

Sixteen articles reported telehealth to be satisfactory to parents (Criss, 2013; Crutchley & Campbell, 2010; Drabarek et al., 2022; Frederick et al., 2020; Gallagher, 2004; Green et al., 2023; Rose et al., 2000; Svendsen et al., 2023; Zhang et al., 2023), children (Frederick et al., 2020; Moirangthem & Ojha, 2022; Stephan et al., 2016; Zhang et al., 2023), providers (Drabarek et al., 2022; Erickson et al., 2021; Hines et al., 2015; Reupert, Greenfeld, et al., 2022; Stephan et al., 2016), and educators (Bloomfield et al., 2020; Crutchley & Campbell, 2010; Drabarek et al., 2022; Fischer, Dart, et al., 2017; Stephan et al., 2016). Authors reported that children were engaged and enjoyed the telehealth services (Erickson et al., 2021; Fairweather et al., 2017; Lam et al., 2021; Lincoln et al., 2015). There was also a strong complement of articles (*n* = 11) reporting that services were effective and equivalent to in-person alternatives (Bice-Urbach & Kratochwill, 2016; Boisvert & Hall, 2019; Bradford et al., 2018; Fischer, Collier-Meek, et al., 2017; Fischer, Dart, et al., 2019; Fischer, Dart, et al., 2017; Gabel et al., 2013; Grogan-Johnson et al., 2011; Langbecker et al., 2019; Lee et al., 2017; Machalicek et al., 2009). However, not all articles were clear that telehealth was equivalent. Some programs described continuing to collect data to monitor and adapt telehealth services to ensure they are meeting students’ needs and goals (Hall, 2022; Kriechman et al., 2010). There were mixed responses as to whether or not technology was easy to use (Gabel et al., 2013; Hyde et al., 2022; Lincoln et al., 2015) or that technology issues, such as reduced sound quality and unreliable internet connections, interfered with the service delivery (Fairweather et al., 2017; Grogan-Johnson et al., 2010; Norman et al., 2021). Two articles reported that children preferred in person services to telehealth (Moirangthem & Ojha, 2022; Svendsen et al., 2023) and two articles reported that providers would not choose to use telehealth in the future (Lam et al., 2023; Reupert, Greenfeld, et al., 2022). Finally, seven of the articles discussed that the telehealth model met a need such as access to services in rural or other underserved communities (Bradford et al., 2018; Cox et al., 2000; Frederick et al., 2020; Lincoln et al., 2015; Marrapese et al., 2021; Miller et al., 2002; Shahidullah et al., 2023).

#### Adopter Categories

Prior to the pandemic, in some circumstances, telehealth had not yet been adopted (Erickson et al., 2021; Hall, 2022; Rortvedt & Jacobs, 2019) or was in the innovator and early adopter stages of implementation where few providers had adopted telehealth (Abbott-Gaffney & Jacobs, 2020; Schultz et al., 2018; Tucker, 2012). Those who had adopted prior to the pandemic would be considered more venturesome and potentially role models for others (Rogers, 2003; Zhang et al., 2023). Prior to the pandemic, the rate of adoption was slow (Bolden & Grogan-Johnson, 2022) and those who were hesitant were likely pushed towards adoption with the pandemic restrictions (Farmer et al., 2020). However, one study noted telehealth use by 56% of providers in March 2020 (onset of pandemic restrictions) and the remainder by June 2020 (Lam et al., 2023). Additionally, Berger and colleagues found that 16% (laggards) did not do psychological assessments during the COVID-19 pandemic (2023). The rate of adoption of new technology was described as being dependent on funding and system commitment (Cox, 1998).

### Social System

The social system, the fourth category of DOI, involves how a group of people problem solve to meet a goal and is influenced by the norms and structure for behaviour (Rogers, 2003). Included in the social system category are the concepts of influencers, innovation decisions (optional, collective, authority, or contingent decisions), and consequences of innovations whether desirable or undesirable, direct or indirect, and anticipated or unanticipated. The social system was supportive when: (1) administrators fostered a positive telehealth culture (Bradford et al., 2018; Erickson et al., 2021; Fairweather et al., 2017; Frederick et al., 2020; Hyde et al., 2022; McLellan et al., 2017), (2) e-helpers were available (Boisvert et al., 2012; Bradford et al., 2018; Criss, 2013; Crutchley & Campbell, 2010; Gabel et al., 2013; Grogan-Johnson et al., 2011; Hines et al., 2015; Lee et al., 2017; Lincoln et al., 2015; McLellan et al., 2017; Stephan et al., 2016), and (3) guidelines were in place (Bice-Urbach & Kratochwill, 2016; Bolden & Grogan-Johnson, 2022; Farmer et al., 2020; Grogan-Johnson, 2021; Hall, 2022; Lowman & Kleinert, 2017; Lundblom et al., 2022; Stephan et al., 2016). Decisions were frequently made as authority decisions by administrators (Crutchley & Campbell, 2010; Fairweather et al., 2017; Fischer, Dart, et al., 2017; Frederick et al., 2020; Tucker, 2012; Walker et al., 2021) or due to the pandemic restrictions (Frederick et al., 2020; Hall et al., 2021; Hall, 2022; Hyde et al., 2022; Lundblom et al., 2022; Reupert, Schaffer, et al., 2022). The influencers of the social system, as well as the indirect, anticipated, and unanticipated consequences of the innovation of telehealth in schools were minimally reported within the literature.

Telehealth had desirable consequences, such as student progress towards behavioural and language goals (Bice-Urbach & Kratochwill, 2016; Boisvert et al., 2012; Cox et al., 2000; Criss, 2013; Crutchley & Campbell, 2010; Fischer, Bloomfield, et al., 2019; Fischer, Dart, et al., 2017; Gabel et al., 2013; Gallagher, 2004; Grogan-Johnson et al., 2010; Grogan-Johnson et al., 2011; Lam et al., 2021; Langbecker et al., 2019; Lee et al., 2017; Lincoln et al., 2015; Miller et al., 2002; Shahidullah et al., 2022; Stormshak et al., 2019; Walker et al., 2021), efficiency and increased access of services (Bloomfield et al., 2020; Bradford et al., 2018; Daftary, 2021; Fairweather et al., 2017; Hyde et al., 2022; Norman et al., 2021; Shahidullah et al., 2022), and increased access to parents (Crutchley & Campbell, 2010; Daftary, 2021; Erickson et al., 2021; Fairweather et al., 2017; Gallagher, 2004; Hyde et al., 2022; Kriechman et al., 2010; Shahidullah et al., 2022; Walker et al., 2021). Telehealth also had undesirable consequences, such as increased stress, time, and fatigue of providers (Erickson et al., 2021; Hall, 2022; Hines et al., 2015), decreased student attendance and attention challenges (Bradford et al., 2018; Criss, 2013; Erickson et al., 2021; Hall, 2022; Lincoln et al., 2015), and decreased collaboration (Fischer, Collier-Meek, et al., 2017; Grogan-Johnson et al., 2011; Stephan et al., 2016), that were also direct consequences of telehealth implementation.

#### Influencers

Three articles addressed the concept of influencers. Providers sought more information and learned about telehealth through online communities through their professional associations, Facebook, and YouTube (Grogan-Johnson, 2018, 2021). Site coordinators also played a role in influencing educators, parents, students, providers, and organizations to participate in telehealth through their role as coordinators (Zhang et al., 2023).

#### Innovation Decision

Seven articles reported an authority decision was made due to the COVID-19 pandemic restrictions (Frederick et al., 2020; Hall et al., 2021; Hall, 2022; Hyde et al., 2022; Lam et al., 2023; Lundblom et al., 2022; Reupert, Schaffer, et al., 2022). An additional six authority decisions were made by school administrators due to their control over budgets, and allocation of equipment, space, and personnel (Crutchley & Campbell, 2010; Fairweather et al., 2017; Fischer, Dart, et al., 2017; Frederick et al., 2020; Tucker, 2012; Walker et al., 2021). Optional decisions (i.e., decisions made by individuals) were made by either families (Lundblom et al., 2022; Marrapese et al., 2021) or providers (Farmer et al., 2020; Walker et al., 2021). A contingent decision (i.e., a decision to adopt or reject made only after a prior decision) was discussed in one article wherein an authority decision had been made due to COVID-19 restrictions but subsequently an individual or collective decision could be made by providers based on student suitability and families who consent to telehealth (Hall, 2022).

#### Desirable Consequences

The desirable consequences of implementation of telehealth in school services, were related to the child’s skills, the providers’ efficiency, an increase in access for health services, and improved parent engagement. The students were described to have made progress towards their goals, with 23 of the articles reporting on goals relating to behaviour and/or language (Bice-Urbach & Kratochwill, 2016; Boisvert et al., 2012; Cox et al., 2000; Craig et al., 2023; Criss, 2013; Crutchley & Campbell, 2010; Fischer, Bloomfield, et al., 2019; Fischer, Dart, et al., 2017; Gabel et al., 2013; Gallagher, 2004; Green et al., 2023; Grogan-Johnson et al., 2010; Grogan-Johnson et al., 2011; Hämäläinen et al., 2023; Lam et al., 2021; Langbecker et al., 2019; Lee et al., 2017; Lincoln et al., 2015; Miller et al., 2002; Shahidullah et al., 2022; Stormshak et al., 2019; Svendsen et al., 2023; Walker et al., 2021). Students were also reported to have positive engagement with telehealth (Bradford et al., 2018; Green et al., 2023; Hyde et al., 2022; Martinez et al., 2023; Norman et al., 2021; Reupert, Greenfeld, et al., 2022; Svendsen et al., 2023; Walker et al., 2021) and increased school attendance and school engagement (Fairweather et al., 2017; Hämäläinen et al., 2023). The technology allowed for the students to turn off their cameras and use the chat function for increased student comfort in participating (Martinez et al., 2023). The telehealth services were also more efficient by reducing travel and increasing flexibility for scheduling (Boisvert & Hall, 2019; Erickson et al., 2021; Fischer, Dart, et al., 2017; Hyde et al., 2022; Martinez et al., 2023; Reupert, Greenfeld, et al., 2022; Rose et al., 2000; Stephan et al., 2016; Svendsen et al., 2023). These efficiencies can lead to increased access and availability of health services (Bloomfield et al., 2020; Bradford et al., 2018; Daftary, 2021; Fairweather et al., 2017; Hyde et al., 2022; Norman et al., 2021; Reupert, Greenfeld, et al., 2022; Shahidullah et al., 2022; Shahidullah et al., 2023; Svendsen et al., 2023). Finally, 13 studies reported an increase in inclusion of parents in services and the ability to build a relationship with parents using telehealth for communication (Crutchley & Campbell, 2010; Daftary, 2021; Drabarek et al., 2022; Erickson et al., 2021; Fairweather et al., 2017; Gallagher, 2004; Hyde et al., 2022; Kriechman et al., 2010; Reupert, Greenfeld, et al., 2022; Shahidullah et al., 2022; Svendsen et al., 2023; Walker et al., 2021; Zhang et al., 2023).

#### Undesirable Consequences

Telehealth is not for everyone. Providers reported feelings of stress and anxiety related to utilizing and troubleshooting technology challenges (Erickson et al., 2021; Hall, 2022; Hines et al., 2015). Providers also reported that telehealth takes more time to schedule and set up, or to use to build relationships, and providers were exhausted following telehealth sessions (Erickson et al., 2021; Hines et al., 2015; Lam et al., 2023; Zhang et al., 2023). Some students also found telehealth difficult at times and were noted to be frustrated (Criss, 2013), not engaged (Berger et al., 2023; Bradford et al., 2018; Lincoln et al., 2015; Martinez et al., 2023; Reupert, Greenfeld, et al., 2022; Svendsen et al., 2023), distracted (Erickson et al., 2021; Lam et al., 2023), and had increased absenteeism (Hall, 2022). Additionally, telehealth was reported to be less effective (Berger et al., 2023; Bolden & Grogan-Johnson, 2022; Craig et al., 2023; Gabel et al., 2013; Lam et al., 2023; Lam et al., 2021; Reupert, Greenfeld, et al., 2022), and some providers lowered their expectations during telehealth sessions (Daftary, 2021). Without physical presence within the school, providers found it challenging to debrief with parents (Drabarek et al., 2022), understand the school culture (Stephan et al., 2016), and to collaborate with educators (Fischer, Collier-Meek, et al., 2017; Grogan-Johnson et al., 2011). Finally, it was perceived that some students and families had concerns about privacy when using telehealth (Berger et al., 2023; Daftary, 2021; Martinez et al., 2023; Reupert, Greenfeld, et al., 2022; Stephan et al., 2016).

One article identified concerns with the real or perceived discrimination around who is provided with telehealth versus who is provided with in-person services (Hall, 2022). For instance, if one school is selected as a provider’s home school where they have their workspace and provide in-person services to students attending that school, other schools on their caseload are then designated telehealth schools. The potential for discrimination exists in how the home school was selected and if students are accessing ideal service delivery for their unique needs (Hall, 2022).

#### Direct/Indirect and Anticipated/Unanticipated Consequences

The desirable and undesirable consequences of telehealth were also considered direct, immediate consequences with only two exceptions. Direct consequences are those that result in an immediate change to an individual or social system irrespective of their desirability (Rogers, 2003). The few additions include statements of students’ enjoyment and language skill development, and parents’ view about the amount of therapy (Lam et al., 2021). Additionally, one article commented neutrally on the fact that telehealth is more frequently provided in individual pull-out sessions versus small group pull-out sessions in-person (Gabel et al., 2013); however, another article presented this positively as it increased individual attention (Crutchley & Campbell, 2010). Also, one article reported an inability to meet individually with recipients following a group session but were able to mitigate this by providing phone and email availability (Drabarek et al., 2022). There were only three articles that presented indirect, secondary consequences (Bolden & Grogan-Johnson, 2022; Daftary, 2021; Shahidullah et al., 2022) and no articles discussed either anticipated or unanticipated consequences of telehealth.

#### Supportive Social System

Additional data were extracted pertaining to the social system. These demonstrated that telehealth was successful when the school health provider had additional social supports. The school district and administration needed to foster a culture and environment for telehealth (Berger et al., 2023; Bradford et al., 2018; Erickson et al., 2021; Fairweather et al., 2017; Frederick et al., 2020; Hyde et al., 2022; McLellan et al., 2017). Providers noted that they relied on e-helpers to supervise the students, support the technological aspects, and facilitate communication with other team members (Boisvert et al., 2012; Bradford et al., 2018; Criss, 2013; Crutchley & Campbell, 2010; Gabel et al., 2013; Grogan-Johnson et al., 2011; Hines et al., 2015; Lee et al., 2017; Lincoln et al., 2015; McLellan et al., 2017; Stephan et al., 2016; Zhang et al., 2023). Providers expressed a need to be planned and deliberate when collaborating with educators and parents given the virtual nature of the services (Brunson McClain et al., 2021; Drabarek et al., 2022; Grogan-Johnson, 2018; Lincoln et al., 2015; Lundblom et al., 2022; Tambyraja et al., 2021). Additionally, government policies and professional practice guidelines can influence telehealth delivery in schools depending on local, regional, and national policies on delivery and reimbursement of services (Bice-Urbach & Kratochwill, 2016; Bolden & Grogan-Johnson, 2022; Farmer et al., 2020; Grogan-Johnson, 2021; Hall, 2022; Lowman & Kleinert, 2017; Lundblom et al., 2022; Stephan et al., 2016).

### Other Barriers and Facilitators

Technology access issues and internet connectivity were by far the most frequently reported barriers to telehealth delivery in school (Bice-Urbach & Kratochwill, 2016; Boisvert & Hall, 2019; Brunson McClain et al., 2021; Craig et al., 2023; Crutchley & Campbell, 2010; Erickson et al., 2021; Fairweather et al., 2017; Farmer et al., 2020; Fischer, Bloomfield, et al., 2019; Fischer et al., 2016; Frederick et al., 2020; Green et al., 2023; Grogan-Johnson, 2021; Hall, 2022; Lowman & Kleinert, 2017; Martinez et al., 2023; Stephan et al., 2016; Walker et al., 2021) and inversely were reported as a facilitator when reliable internet, software, and hardware was available (Berger et al., 2023; Bice-Urbach & Kratochwill, 2016; Boisvert et al., 2012; Drabarek et al., 2022; Erickson et al., 2021; Fischer, Dart, et al., 2019; Fischer, Dart, et al., 2017; Fischer et al., 2018; Grogan-Johnson, 2021; Grogan-Johnson et al., 2011; Hall, 2022; Hines et al., 2015; Lowman & Kleinert, 2017; Marrapese et al., 2021; Nelson et al., 2023; Tambyraja et al., 2021). Additionally, lack of training of providers, teachers, and parents when supporting children during telehealth sessions was also reported as a barrier to implementing telehealth in schools (Fischer, Dart, et al., 2019; Hall et al., 2021; Lam et al., 2023; Lowman & Kleinert, 2017; Tambyraja et al., 2021). Similarly, training was reported as a facilitator when provided to providers prior to telehealth implementation (Abbott-Gaffney & Jacobs, 2020; Berger et al., 2023; Boisvert & Hall, 2019; Erickson et al., 2021; Hall et al., 2021; Hall, 2022; Hines et al., 2015; Lam et al., 2023; Lundblom et al., 2022; Norman et al., 2021; Schultz et al., 2018).

There were some provider attributes that also were identified as facilitators, such as empathy (Daftary, 2021), and a willingness to try new things, flexibility, and organization (Hines et al., 2015). Some providers indicated a need for time to create, or obtain access to, resources for virtual delivery (Berger et al., 2023; Daftary, 2021; Grogan-Johnson, 2021; Hines et al., 2015; Murphy et al., 2021). Access to funds to implement and be reimbursed for telehealth was also a facilitator (Grogan-Johnson, 2018; Kriechman et al., 2010; Nelson et al., 2023; Rortvedt & Jacobs, 2019; Shahidullah et al., 2022) and conversely a barrier when funding was lacking (Crutchley & Campbell, 2010; Daftary, 2021; Fairweather et al., 2017; Fischer, Bloomfield, et al., 2019; Murphy et al., 2021; Tucker, 2012). Finally, guidelines and legal requirements, such as concerns for privacy and ethics, were additional factors that influence telehealth implementation (Farmer et al., 2020; Fischer, Bloomfield, et al., 2019; Fischer, Dart, et al., 2019; Fischer, Dart, et al., 2017; Grogan-Johnson, 2018; Lam et al., 2023; Marrapese et al., 2021; Reupert, Greenfeld, et al., 2022; Reupert, Schaffer, et al., 2022; Rortvedt & Jacobs, 2019). One study specifically noted that technology develops faster than the policies and laws to support the technology (Fischer, Bloomfield, et al., 2019). Apart from those identified using DOI theory, barriers and facilitators to telehealth implementation were technology, training, time, funding, and guideline resources depending on their availability.

## Discussion

This review aimed to describe the factors that influence telehealth use in schools by health professionals. The results spanned the years from 1998–2023, which represented data collected both prior to and during the COVID-19 pandemic. Therefore, when summarizing the extracted data, the authors considered if there were potential differences between these time periods. However, the extracted implementation factors remained quite stable across time. This stability is evident throughout the narrative presentation of the results as well as visually in Table 2 whereby the publication years for citations in support of the results included literature published before, during, and following the pandemic. This suggests that the implementation factors identified in this study are not idiosyncratic or uniquely reflective of telehealth implementation during the COVID-19 pandemic.

Using DOI theory to guide our analysis, we observed that many factors influence when and if telehealth is implemented into school-based health services. Sixty-six articles included data about more than one of the four main categories within the DOI theory, with some articles addressing as few as two and others as many as 13 of the DOI subcategories, which we extracted. Given this variation across articles and DOI categories, we offer a synthesis here of key findings formulated as recommendations for professional practice as well as considerations for implementation arising from our application of DOI theory.

### Recommendations for Professional Practice

This scoping review revealed several key elements required to implement telehealth in school-based health services, including necessary personnel, training and supports, material resources, and policies. The identification of training and materials as key elements is also supported by a scoping review of telehealth provided by SLPs within and outside the school setting to children residing in rural areas that identified training and equipment (software and internet connectivity) as the most frequently reported facilitators for telehealth implementation (Campbell et al., 2020). Managers who support staff initiatives are also important in the implementation of a novel innovation due to their ability to allocate resources, funding, and personnel time to the initiative (Kristensen et al., 2016).

As identified in the time subcategory of implementation and the social system category of DOI, necessary personnel included having an e-helper available for on-site supervision of the student, technology trouble shooting, and communication with educators (Boisvert et al., 2012; Bradford et al., 2018; Criss, 2013; Crutchley & Campbell, 2010; Gabel et al., 2013; Grogan-Johnson et al., 2011; Hines et al., 2015; Lee et al., 2017; Lincoln et al., 2015; McLellan et al., 2017; Stephan et al., 2016; Zhang et al., 2023). The e-helper was also instrumental in setting up the technology and in getting the student to the telehealth service room for the appointment time (Fairweather et al., 2017; McLellan et al., 2017; Stephan et al., 2016). We learned in our primary scoping review that e-helpers can be therapy assistants, educational assistants, parents, clinical students, or teachers, depending on the availability of personnel (Knobl et al., 2024). The cost of the e-helper and their flexibility of scheduling is important to consider when planning a telehealth program.

Training the e-helper in best practices, rapport building, and technology skills also was important and was identified in the implementation subcategory of time. Telehealth providers found that e-helpers needed to be trained in setting up and troubleshooting the technology to be used during services (Boisvert & Hall, 2019; Crutchley & Campbell, 2010; Fischer, Bloomfield, et al., 2019; Fischer, Dart, et al., 2019; Gabel et al., 2013; Grogan-Johnson et al., 2011; Schultz et al., 2018). The e-helper training also entailed explaining their role during the service delivery (Gabel et al., 2013; Grogan-Johnson et al., 2011; Nelson et al., 2023; Shahidullah et al., 2023). The role varied and can include adjusting camera angles, printing and gathering materials the student needed during telehealth services, and facilitating collaboration with the classroom teacher by relaying messages and telehealth activities completed during a session. Given the varied roles the e-helper can take on, the training needed by the e-helper varies.

Health providers also reported needing training as per the subcategories of complexity (innovation) and knowledge (time). Their training entailed technology set up, use, and troubleshooting skills (Abbott-Gaffney & Jacobs, 2020; Boisvert & Hall, 2019; Bolden & Grogan-Johnson, 2022; Craig et al., 2023; Crutchley & Campbell, 2010; Erickson et al., 2021; Grogan-Johnson, 2021; Hall, 2022; Moirangthem & Ojha, 2022) but in addition delved into relationship building with both the student and the educators given the telehealth method for service delivery (Abbott-Gaffney & Jacobs, 2020; Frederick et al., 2020; Hines et al., 2015). Similarly, technology skills and training are identified in the broader telehealth implementation research as being required for success (Borges Do Nascimento et al., 2023; Saigí-Rubió et al., 2022). The telehealth training provided and received was not well described in the literature; however, implementation research is able to further describe components of successful education programs.

Implementation researchers employ many different educational strategies to increase knowledge of health care providers. Clinical guidelines are one educational strategy traditionally employed in health care settings to share knowledge and guidance to health care providers (Grol & Grimshaw, 2003). Clinical guidelines have mixed effects with increased effectiveness when implemented for acute conditions, with quality evidence, compatible with existing practices, requiring fewer new skills, and necessitating less organizational change (Grol & Grimshaw, 2003). Unfortunately, school-based services do not meet many of these characteristics. First, school-based services are often provided for chronic conditions. Second, although quality of evidence was not assessed for this scoping review, many of the studies were pilot and survey studies. Additionally, review studies addressing telehealth more broadly have confirmed that the methodological quality of the evidence is low to critically low (Borges Do Nascimento et al., 2023; Saigí-Rubió et al., 2022). Third, our assessment of compatibility within this scoping review identified both similarities and differences between telehealth and existing in-person services. Fourth, training for new skills needed for telehealth delivery was identified as a barrier to implementation. And finally, organizational change is required for implementation of telehealth within the school context. Given these characteristics, clinical practice guidelines are not likely to result in change when implementing telehealth in schools. Emails, meetings, newsletters, and conference attendance also do not lead to change nor support for healthcare innovation implementation (Kristensen et al., 2016).

Implementation researchers encourage the use of a plan for interactive education approaches that involve continuous engagement in education, use of evidence-based information, and feedback (Grol & Grimshaw, 2003). The data extracted using DOI theory related to trialability (*n* = 11) and observability (*n* = 2) were limited but this may support the notion of interactive approaches to education. Similarly, the influencers (*n* = 3) and communication channels (*n* = 7) subcategories also resulted in minimal data extractions, where these subcategories may support the education and feedback about the innovation implementation. The influencers subcategory did capture that health providers were seeking feedback about telehealth from online communities (Grogan-Johnson, 2018, 2021). Further research is needed to better understand the training and education methods best suited to support telehealth implementation in schools.

Material resources for telehealth included the students and providers having access and funding for reliable technology that met the needs of the service. These were reported in the social system category and the subcategories of compatibility (innovation) and implementation (time) of DOI. The predominant issue with technology was internet connectivity. Securing a stable internet connection at the school is important and can influence sound quality (Fairweather et al., 2017; Hines et al., 2015), productive use of session time (Daftary, 2021; Fairweather et al., 2017; Farmer et al., 2020), and provider and student stress and engagement (Bradford et al., 2018; Erickson et al., 2021; Tambyraja et al., 2021). Most of the included articles made use of videoconferencing for telehealth (Knobl et al., 2024). Access to hardware, such as a tablet or laptop with webcam for videoconferencing was not frequently a barrier to implementation when the student was joining a telehealth session from the school.

Another important material resource, as identified in the subcategory of compatibility of the innovation, was having access to assessment tools that could be administered using telehealth. While the literature supported use of assessments that were observational or based on parent, teacher, or student report, caution is needed when conducting standardized assessments if they are not yet standardized for telehealth administration. Standardized assessments were discussed as having many more obstacles to telehealth implementation to ensure they are valid and reliable when administered using telehealth modalities (Berger et al., 2023; Brunson McClain et al., 2021; Farmer et al., 2020; Tucker, 2012).

Finally, policies were identified within the social system category of DOI as well as the barriers and facilitators category as being needed to support telehealth delivery. These policies included professional standards, ethics, privacy, and funding for reimbursement. Students, families, and providers reported concerns with privacy when accessing telehealth at home or in a shared location of the school (Daftary, 2021; Fischer, Bloomfield, et al., 2019; Fischer, Dart, et al., 2019; Hall, 2022; Lam et al., 2023; Lundblom et al., 2022; Reupert, Greenfeld, et al., 2022; Reupert, Schaffer, et al., 2022; Rortvedt & Jacobs, 2019; Stephan et al., 2016). A private space is recommended for telehealth implementation. Professional standards vary between different professions and geographic locations and were frequently updated during the COVID-19 pandemic, as were reimbursement funding policies. Procedures and funding clarity were both identified as requirements in systematic review studies of telehealth implementation in various health care settings across Europe and in rural Australia (Bradford et al., 2016; Saigí-Rubió et al., 2022). Providers need to remain aware and up to date with their professional and local policies and standards related to telehealth.

### Theoretical Recommendations for Implementation Planning

While DOI theory successfully captured a wide array of factors affecting implementation of telehealth in school-based health services, we did observe one notable limitation. Specifically, the theory has a positive lens towards adoption, meaning it does not explicitly address failed adoption or barriers to implementation. Rogers identifies this as the ‘pro-innovation bias’ and as a criticism of diffusion research (2003). In our study, we adapted our analysis to include additional categories beyond the DOI categories to capture these negative factors.

The NASSS (nonadoption, abandonment, scale-up, spread, and sustainability) is one alternative implementation theory that includes nonadoption. The NASSS technology implementation framework includes seven domains and 13 questions intended to guide balanced discussions to generate ideas for implementation (Greenhalgh et al., 2017). This model may be helpful during the design phase of planning to use telehealth in school-based practice. Identifying barriers is an important factor when creating implementation plans. Identifying barriers, linking barriers to intervention, use of theory, and engaging users’ feedback were the four tasks found to be in agreement within a systematic review of 15 papers looking at interventions designed for behavioural change of healthcare providers (Colquhoun et al., 2017).

Another aspect of planning for implementation of telehealth, is looking to both the strategic and operational strategies when planning, managing, and sustaining change (Kho et al., 2020). Using scoping review methodology, Kho and colleagues (Kho et al., 2020) identified 16 change management strategies related to implementing telehealth and created a framework for telehealth implementation. Several change management strategies from their framework aligned with the findings of our review such as assessing compatibility, assigning roles (e-helper), leadership support (social system), ensuring adequate resources (access to equipment), training and education, development of processes and protocols, monitoring change (confirmation), maintaining flexibility (re-invention), and evaluating change (consequences of innovation). Additionally, communication and identification of champions are two change management strategies identified by Kho et al.’s framework that were minimally present in the literature included in our scoping review (7 of 70 articles addressed communication channels and 3 of 70 identified influencers). Finally, strategies such as conducting a needs assessment, engaging invested parties, developing a vision, and facilitating ownership were not directly addressed in our scoping review using the DOI theory. It may be beneficial to explore these areas in future research for improved implementation of telehealth in school-based practice.

### Recommendations for Future Research

By utilizing DOI theory to categorize the factors affecting implementation of telehealth in schools, it became clear that some elements of diffusion are not well reported in the current literature. It is possible that publication standards could explain this. For example, authors may be less likely to publish about indirect consequences of innovations as these would likely be secondary outcomes that were not measured in their study (Portney, 2020). Similarly, anticipated and unanticipated consequences are unlikely to be included in publications because for a reader to understand if a consequence was anticipated or unanticipated, the author would have to outline their predictions, which is often only included as a hypothesis for statistical purposes (Portney, 2020). Additionally, some of the categories are likely minimally reported because the included studies were not diffusion studies. These include the adopter categories, the communication channels, and the innovation-decision type. The use of an implementation theory, such as DOI, when designing a research study would likely lead to greater insights about factors that influence the implementation of telehealth in school-based practice.

Future research should focus on adding evidence to describe the effects of trialling and observing telehealth. Many providers have now had the opportunity to trial telehealth during the COVID-19 pandemic; however, there is currently limited research around the effects of trialling telehealth towards consistent implementation. Additional research could also focus on the communication channels and influencers to better understand who and through what channels do providers learn about telehealth to decide, or not, to implement telehealth. Finally, limited data was available about the adopter categories and the innovation-decisions. Such data would inform researchers about who is adopting telehealth and who made the decision, whether optional, collective, or authority, to adopt telehealth. Gaining a more in-depth understanding of these factors that were explored minimally in the research included in this scoping review will give researchers, clinicians, administrators, and policy makers a more complete picture of the diffusion of telehealth in school-based health services.

Future research should also focus on adding the perspective of parents and students in the initial processes related to time such as knowledge, persuasion, and decision. It was noted that the family and student perspective was most commonly sought when evaluating satisfaction with telehealth but was not included in the decision to use telehealth or in the planning of the research or implementation of telehealth. Kho and colleagues (Kho et al., 2020) recommend stakeholder collaboration at all phases of strategic planning when implementing and managing change. Additionally, inclusion of families in research is known to have the following benefits: determining relevance of the innovation, setting priorities, and improving pragmatic components of research such as recruitment and retention (C. Morris et al., 2011). Once included, families and students, key recipients, can advise under what conditions and what services to implement using telehealth.

Further research also is needed to determine the compatibility of standardized assessments for telehealth use. Standardized assessments are developed and tested to ensure they are reliable and valid. A reliable and valid assessment allows a health provider to feel confident that the information they obtain through the assessment is accurate, relevant, and meaningful (Portney, 2020). For instance, Pearson (Pearson, 2023), the publisher of many pediatric and school relevant assessments, states that delivering assessments via telehealth may have varied results based on the role and training of the onsite facilitator, as parents may interfere with testing or may not complete their role as directed. Additionally, the publisher recommends considering the following five areas when adapting assessments for telehealth delivery: audio-video equipment and environment, examiner considerations, examinee considerations, assessment and assessment materials, and other miscellaneous considerations. Pearson also states that not all assessments or subtests are recommended for telehealth as some are more complicated to administer via telehealth. Finally, they recommend that health providers use their professional judgement, abide by their professional guidelines, and document any adaptations made to the standardized procedures, including use of telehealth delivery.

Additionally, although there were several studies that addressed effectiveness of telehealth, more research is needed to increase the robustness of the research in this area as many of the studies included small sample sizes, ideal conditions (available technology, reliable internet, and school support), or were survey-based studies. Implementation studies are needed to develop the policies and procedures necessary to support telehealth implementation. Finally, cost-effectiveness research is warranted. Many health providers reported a time and workload management savings; however, the technology for telehealth and e-helpers to support on-site implementation can be costly resources. Research is needed to quantify the cost savings and expenditures for telehealth implementation.

### Study Limitations

We limited our scoping review to include only peer-reviewed articles and only those written in English, which may have limited the breadth of information captured by our review. Additionally, scoping review methods do not require quality appraisal; therefore, any reports of effectiveness of telehealth in schools should be taken with caution (Peters, Godfrey, et al., 2020). The purpose of this review was to describe the literature pertaining to the factors that influence telehealth use in schools and not to determine the effectiveness of telehealth in schools. Perceived effectiveness was reported for both the category of confirmation and desirable consequences; however, such reports were often based on perspectives of providers, educators, students, and/or parents. To determine the efficacy of telehealth services in schools or how these services compare to in-person services, randomized superiority, non-inferiority, or equivalency trials are needed (Portney, 2020).

## Conclusions

This scoping review revealed that there are many factors that when combined, lead to implementation of telehealth in schools. For providers and schools interested in implementing telehealth, the most salient identified factors include having an e-helper available (time and social system), providing training for e-helpers and providers (innovation and time), access to reliable technology (innovation, time, and social system), and having policies to support ethical, legal use and reimbursement of telehealth (social system). Future research is needed to confirm the effectiveness and cost-efficacy of telehealth use in schools to support the development of needed policies and procedures. Additional implementation research is warranted to fill gaps in the literature identified by this review, such as identifying the secondary consequences of implementing telehealth, describing the role of trialability and observability of telehealth, describing the communication channels through which an individual learns about telehealth, and exploring the adopter categories of who adopts telehealth quickly and slowly. With the increase in use of telehealth due to provider shortages, limited access to services in rural areas, and the COVID-19 pandemic restrictions, it is important to determine how to best implement telehealth practices moving forward when working with students in schools.

## Figures and Tables

**Table 1:** Data Extraction Categories

Innovation		

	Characteristics	Relative Advantage, Compatibility, Complexity, Trialability, Observability, Re-invention
	Other	

Communication Channels		

Time		

	Innovation-decision process	Knowledge, Persuasion, Decision, Implementation, Confirmation
	Innovativeness and adopter categories	Innovators, Early Adopters, Early Majority, Late Majority, Laggards
	Rate of Adoption	
	Other	

Social System		

	Influencers	
	Innovation decisions	Optional, Collective, Authority, Contingent
	Consequences of the innovation	Desirable, Undesirable, Direct, Indirect, Anticipated, Unanticipated
	Other	

Other Barriers		
Other Facilitators		
Other Factors		

**Table 2:** Subcategories Extracted Per Article

Article	Innovation							C.C.	Time												Social System													Other barriers	Other facilitators	Other factors
	Characteristics of Innovation								Innovation-Decision					Adopter Categories							Innovation-Decision							Consequences								
	Relative advantage	Compatibility	Complexity	Trialability	Observability	Re-invention	Related to innovation	Communication Channels	Knowledge	Persuasion	Decision	Implementation	Confirmation	Innovators	Early Adopters	Early Majority	Late Majority	Laggards	Rate of adoption	Related to time	Influencers	Optional	Collective	Authority	Sequential	Contingent	Desirable	Undesirable	Direct	Indirect	Anticipated	Unanticipated	Related to social system			
Abbott-Gaffney & Jacobs, 2020	X						X		X	X										X															X	
Berger et al., 2023				X							X	X						X															X	X	X	X
Bice-Urbach & Kratochwill, 2016	X											X	X														X		X				X	X	X	
Bloomfield et al., 2020	X		X										X														X									
Boisvert et al., 2012	X	X				X			X		X		X														X		X				X		X	
Boisvert & Hall, 2019	X	X	X							X		X	X														X		X					X	X	
Bolden & Grogan-Johnson, 2022	X	X							X										X									X	X	X			X			
Bradford et al., 2018													X														X	X	X				X	X		X
Brunson McClain et al., 2021	X	X	X				X					X																					X	X		X
Cox, 1998	X	X											X							X																
Cox et al., 2000	X						X			X			X							X							X		X							
Craig et al., 2023	X		X										X														X	X						X	X	X
Criss, 2013	X	X											X														X	X	X				X			
Crutchley & Campbell, 2010								X		X			X											X			X		X				X	X		X
Daftary, 2021							X				X		X														X	X	X	X			X	X	X	X
Drabarek et al., 2022	X	X				X							X														X	X	X						X	X
Erickson et al., 2021		X			X		X		X	X		X	X							X							X	X	X				X	X	X	X
Fairweather et al., 2017	X	X									X	X												X			X	X	X				X	X	X	
Farmer et al., 2020		X	X				X				X								X			X		X									X	X		X
Fischer et al., 2016	X	X		X							X	X	X																					X		X
Fischer, Dart, et al., 2017	X											X	X											X			X		X					X	X	X
Fischer, Collier-Meek, et al., 2017													X														X	X	X							
Fischer et al., 2018	X	X	X				X																										X		X	
Fischer, Dart, et al., 2019	X												X																					X	X	X
Fischer, Bloomfield, et al., 2019	X	X					X			X		X	X														X		X				X	X		X
Frederick et al., 2020	X	X	X			X				X		X	X											X			X		X				X	X	X	X
Gabel et al., 2013	X											X	X														X	X	X				X			
Gallagher, 2004	X	X											X														X		X							
Green et al., 2023	X												X														X							X		
Grogan-Johnson et al., 2010													X														X		X							
Grogan-Johnson et al., 2011		X					X					X	X														X	X	X				X		X	
Grogan-Johnson, 2018		X	X	X	X						X										X												X	X	X	X
Grogan-Johnson, 2021	X	X	X	X		X	X		X	X											X		X										X	X	X	X
Hall et al., 2021		X		X						X			X											X										X	X	X
Hall, 2022	X		X	X		X	X	X	X	X	X	X	X							X				X	X		X						X	X	X	X
Hamalainen et al., 2023																											X			X						
Hines et al., 2015							X			X		X	X															X					X		X	X
Hyde et al., 2022	X					X					X		X											X			X	X	X				X		X	
Kriechman et al., 2010													X														X		X						X	X
Lam et al., 2023			X	X				X			X		X						X					X				X						X	X	X
Lam et al., 2021										X			X														X	X	X							X
Langbecker et al., 2019	X												X														X		X							
Lee et al., 2017	X											X	X														X						X			
Lincoln et al., 2014												X	X														X	X	X				X			
Lowman & Kleinert, 2017	X							X	X	X	X	X	X																				X	X	X	
Lundblom et al., 2022		X	X																			X		X									X			X
Machalicek et al., 2009	X												X																							
Marrapese et al., 2021	X	X				X						X	X							X		X	X				X		X				X		X	X
Martinez et al., 2023													X														X	X	X					X		
McLellan et al., 2017	X	X																															X			
Miller et al., 2002	X												X														X		X							
Moirangthem & Ojha, 2022			X								X																									
Murphy et al., 2021											X		X																					X	X	
Nelson et al., 2023	X	X	X	X							X	X															X		X					X		X
Norman et al., 2021								X					X														X		X						X	
Pahwa et al., 2021	X									X																										X
Reupert, Greenfeld, et al., 2022			X	X							X																						X		X	
Reupert, Schaffer, et al., 2022											X		X														X	X	X					X	X	
Rortvedt & Jacobs, 2019	X						X		X	X										X				X												X
Rose et al., 2000	X	X					X		X			X	X																				X			X
Schultz et al., 2018	X	X	X								X									X							X		X					X		
Shahidullah et al , 2022	X					X						X																					X		X	X
Shahidullah et al., 2023	X						X					X	X											X			X		X	X					X	X
Stephan et al., 2016	X	X		X		X	X		X			X	X														X	X					X			X
Stormshak et al., 2019	X	X							X			X	X														X		X				X		X	X
Svendsen et al., 2023	X						X						X														X	X	X							
Tambyraja et al., 2021	X	X						X																									X	X	X	X
Tucker, 2012	X	X	X	X			X		X	X		X	X	X	X									X			X		X					X		
Walker et al., 2021	X	X							X			X										X		X			X	X		X			X			X
Zhang et al., 2023	X	X	X				X	X					X							X	X												X			
Total number articles	22	12	8	5	0	4	8	5	7	6	9	13	23	1	1	0	0	0	1	4	1	3	1	7	0	0	21	10	16	3	0	0	16	9	13	15

## Appendix

### Search Strategy

**Table:**

	Terms (searched as keywords and MeSH when available)
**Concept** (Terms combined with OR)	Telehealth Telepractice Teletherapy Telerehab* Telemedicine Telepsychiatry Telepsychology Telecounselling Telenursing Virtual Care
AND	
**Context** (Terms combined with OR)	School* k-12 k-8 k-6 Student* Elementary school* Secondary school* Middle school* Primary school* High school* School health service* Distance learn* Virtual learn* Virtual education Distance education
AND	
**Population** (Terms combined with OR)	Health professional* Occupational therap* Physiotherap*/physical therap* Speech language patholog*/therap* Dietician* Psycholog* Psychotherap* Social work* Nurs* Rehab* Support service* Health service Health personnel School health service* Allied health

### Sample Medline Search

**Table:**

1	Telehealth.mp.
2	Telepractice.mp.
3	Teletherapy.mp.
4	Exp Telerehabilitation/ or Telerehab*.mp.
5	Telemedicine.mp. or exp Telemedicine/
6	Telepsychiatry.mp.
7	Telepsychology.mp.
8	Telecounselling.mp.
9	Telecounseling.mp.
10	Telenursing.mp. or exp Telenursing/
11	Virtual care.mp.
12	1 or 2 or 3 or 4 or 5 or 6 or 7 or 8 or 9 or 10 or 11
13	School*.mp.
14	Exp Schools/ or schools.mp.
15	k-12.mp.
16	k-8.mp.
17	k-6.mp.
18	Student*.mp. or exp Students/
19	Elementary school*.mp.
20	Secondary school*.mp.
21	High school*.mp.
22	Middle school*.mp.
23	Primary school*.mp.
24	Exp school health services/ or school health service*.mp.
25	Distance learn*.mp.
26	Virtual learn*.mp.
27	Virtual education.mp. or ex Education, Distance/
28	Virtual education.mp.
29	13 or 14 or 15 or 16 or 17 or 18 or 19 or 20 or 21 or 22 or 23 or 24 or 25 or 26 or 27 or 28
30	Exp Health Personnel/ or health professional*.mp.
31	Exp Occupational therapy/ or occupational therap*.mp.
32	Exp Physical therapists/ or physical therap*.mp.
33	Physiotherapy*.mp.
34	Exp Speech-Language Pathology/ or speech language pathology*.mp.
35	Exp Speech Therapy/ or speech language therap*.mp.
36	Speech-language pathology*.mp.
37	Exp Language Therapy/ or speech language therap*
38	Dietician*.mp.
39	Psychology*.mp.
40	Exp Psychology/
41	Exp Psychotherapy/ or psychotherap*
42	Exp social work/ or social work*.mp.
43	Nurs*.mp.
44	Exp Rehabilitation/ or rehab*.mp.
45	Support service*.mp.
46	Exp Health Services/ or health service*.mp. or exp Mental Health Services/
47	Health personnel.mp.
48	Exp School Health Services/ or school health service*.mp.
49	Exp Allied Health Personnel/ or allied health.mp.
50	30 or 31 or 32 or 33 or 34 or 35 or 36 or 37 or 38 or 39 or 40 or 41 or 42 or 43 or 44 or 45 or 46 or 47 or 48 or 49
51	12 and 29 and 50
